# The views and experiences of older people with conservatively managed renal failure: a qualitative study of communication, information and decision-making

**DOI:** 10.1186/s12882-019-1230-4

**Published:** 2019-02-04

**Authors:** Lucy Ellen Selman, Katherine Bristowe, Irene J. Higginson, Fliss E. M. Murtagh

**Affiliations:** 1 Population Health Sciences, Bristol Medical School, University of Bristol, Canynge Hall, 39 Whatley Road, Bristol, BS8 2PS UK; 2 Cicely Saunders Institute of Palliative Care, Policy and Rehabilitation, King’s College London, London, UK; 3 Wolfson Palliative Care Research Centre, Hull York Medical School, University of Hull, Hull, UK

**Keywords:** Qualitative research, Kidney disease, Chronic, Professional-patient relations, Communication, Education, Conservative treatment, Palliative care

## Abstract

**Background:**

Older people with advanced kidney disease require information and support from clinicians when deciding whether to have dialysis or conservative (non-dialysis) care. There is evidence that communication practices, information provision and treatment rates vary widely across renal units. However, experiences of communicating with clinicians among patients receiving conservative care are poorly understood. This evidence is essential to ensure support is patient-centred and equitable. Our aim was to explore views and experiences of communication, information provision and treatment decision-making among older patients receiving conservative care.

**Methods:**

In-depth qualitative interviews were conducted with patients with stage 5 chronic kidney disease from three UK renal units. Purposive sampling captured variation in age, co-morbidity and functional status. Interviews were analysed thematically.

**Results:**

20 patients were interviewed (11 were men; median age 82 (range 69–95)). Participants described positive experiences of communicating with clinicians and receiving information, but also negative experiences involving insensitivity, rushing or ambiguity. Participants reported clinicians omitting/avoiding conversations regarding diagnosis and prognosis, and described what helped and hindered good communication and support. They wanted information about their treatment options and illness, but expressed ambivalence about knowing details of disease progression. Clinicians’ views and recommendations regarding treatment influenced patients’ decision-making.

**Conclusions:**

Older patients report variable quality in communication with clinicians and gaps in the information received. Uncertainty about the disease trajectory and patients’ ambivalence regarding information makes communication particularly challenging for clinicians. Tailoring information to patient preferences and conveying it clearly and sensitively is critical. Renal clinicians require support and training to ensure decision-making support for older patients is patient-centred. Future research should examine how clinicians’ communication practices influence treatment decision-making.

## Background

Each year, more than 3500 UK residents and 55,000 US residents over 65 develop end stage kidney disease (ESKD) and start dialysis [1, 2]. Most travel to a dialysis unit three times each week until they die. While initiating dialysis lengthens life for some older adults, the survival benefit appears small or non-existent in those with comorbidities or frailty [3]. The burden of dialysis and its effect on quality of life also outweigh the benefit of longevity for some patients [4, 5]; hence chosen or medically advised ‘comprehensive conservative kidney care’ [6] is recognised as an acceptable alternative [4, 7]. It comprises all aspects of renal care without preparing for dialysis, in conjunction with a multidisciplinary palliative care approach [6]. Patients receiving conservative care spend less time having treatment and are less likely to be admitted to or die in hospital [8–10].

A major challenge for renal clinicians is supporting and informing people with advanced kidney disease as they negotiate treatment options and decide on future treatment. Clinicians report difficulties conveying the uncertainty surrounding treatment options and judging how much information to share [11]. There is evidence that clinicians’ communication practices are inconsistent: patients from renal units with a more established conservative care pathway are more aware of conservative management, less often believe that dialysis guarantees longevity, and have more often discussed the future with staff [12]. In addition, the format, quality and accessibility of information resources [13] and decision aids [14] vary across renal services, as do patient ratings of the quality of communication and decision-making support [15]. These inconsistencies are associated with unwarranted geographical variation in treatment rates for older adults with ESKD [16–18].

Understanding patients’ experiences of communicating with and receiving information from renal clinicians is crucial to inform clinical practice and staff education, and to ensure equity in the management of ESKD. However, previous qualitative research has predominantly focused on patients’ reasons for opting for conservative care [19–21]. We therefore aimed to explore views and experiences of communication and information provision among patients with ESKD receiving conservative care, and their views of the treatment decision.

## Methods

### Study design

Cross-sectional, in-depth qualitative interviews with patients who were recruited as part of a larger longitudinal study [22].

### Setting

Three renal units at hospitals in London and South-East England: Guy’s Hospital, King’s College Hospital, and East Kent Hospitals. All three renal units had specific conservative (non-dialysis) management services at the time of the study. The London units had dedicated renal palliative care nurse specialists coordinating and providing much of the service, including clinic review and home visits as necessary, with support from nephrology staff as required. The third unit is more rural, and offered more home-based care (including home visits from nephrology staff), telephone support, and integration with primary care and specialist palliative care.

### Sampling

Participants had made a definite decision, in discussion with their nephrology teams, for conservative management and were being conservatively managed at the time of the interview. Inclusion criteria were chronic kidney disease (CKD) stage 5 [23], definite decision for conservative management, and estimated glomerular filtration rate (eGFR) ≤ 15 mL/minute [24] at recruitment into the longitudinal study [22]; qualitative interviews were conducted within three months. Patients who lacked capacity to consent were excluded. Purposive sampling captured variation in age, co-morbidity and functional status. Sampling continued until data saturation, i.e. no new themes were identified in later interviews [25].

### Recruitment

Clinicians gave patients information about the study. FM contacted and recruited interested patients by telephone. Participants gave written informed consent.

### Data collection

An interview topic guide (Table 1) was developed based on study objectives and piloted with three patients (data not included). All interviews were conducted in 2007 by FM, a palliative care clinician trained in in-depth interviewing who was conducting a PhD in Palliative Care. She was not known to the participants, was not involved in their care, and was introduced as an independent researcher. Interviews were audio-recorded, transcribed verbatim and anonymised. Names were changed to age- and ethnicity-specific alternatives. Field notes made during the interview were integrated in the analysis.

**Table 1:** Topic guide

Interview topic	Areas covered
Background	Age
	Social circumstances
	Duration of kidney problem
	Duration of attendance at nephrology department
	Current functional ability
Understanding of illness	Understanding of their kidney disease
Illness impact	How the kidney disease is affecting them, including:• Overall impact on their life • Particular effects – both physical and psychological • What is changing for them • Impact patient perceives on family/carers
	The relative importance of the different factors described [Prompt: the role of symptoms]
	How they see the illness affecting them in the future
Information and decision-making	Experiences of illness information and involvement in healthcare decisions, including: • Factors influencing the experiences • Relative importance of these factors • Anything which might cause a future change in decision • Prompt regarding dialysis decision if not spontaneously introduced

Interviews were conducted according to participants’ preferences, usually (17/20) in their own home. One interview was in a nursing home, and two interviews at a relative’s house, where the participant lived. Where possible, participants were interviewed alone; 7/20 interviews were conducted with a spouse/family member present, either because participants asked for them to be present (3/20), or because there was no alternative living space for family members.

### Analysis

Interviews were analysed using inductive thematic analysis [26], a theoretically flexible approach appropriate in exploratory research [27]. Our research was in the subtle realist paradigm, in which reality is conceptualised as existing objectively, but known only from each individual’s own perspective [28]. Data were managed in NVivo [29], and analysed following these steps: (1) Familiarisation, via detailed reading of transcripts and field notes, and making reflective comments on the nature and content of the interviews (FM). (2) Development of a formal coding index to capture themes and sub-themes in the data. This was an iterative process which involved double coding of three transcripts, additional in-depth coding to meet the aims of the analysis reported here (LES), and discussion across the research team (FM/LES/KB). We used a combination of inductive coding based on close reading of the data and deductive coding based on the study aims. (3) Application of the finalised coding frame to all transcripts (FM/LES). (4) Narrative synthesis of themes, paying attention to patterns in the data and deviant cases (LES). The synthesis was critically reviewed within the research team and refined.

### Ethics

Ethical approval for all research sites was obtained from King’s College Hospital Research Ethics Committee (COREC number 04–03-092, 2006). All participants gave written informed consent.

## Results

### Participants

Twenty-one patients were invited to participate. One declined due to ill health; the remaining 20 were interviewed. Interviews lasted 38–74 min. Participants had a median age of 82 years (range 69–95) and 11 were men (Table 2). Median eGFR at study entry was 12.3 mL/min (range 6.5–14.9 mL/min), median Karnofsky Performance Status Score was 60% (range 40–80%), and median Charlson Comorbidity Index Score was 4 (range 4–8).

**Table 2:** Participants’ clinical characteristics (*n* = 20)

Participants	20
Gender	
Female	11
Male	9
Age	
< 75	2
75–79	5
80–84	8
≥ 85	5
Marital Status	
Married	8
Widowed	9
Single	3
Ethnicity	
White British	18
Afro-Caribbean	1
Other	1
Renal Diagnosis	
Diabetes Mellitus	5
Glomerulo-nephritis	3
Renal vascular disease	2
Unknown	9
Other	1
Karnofsky Performance Status Score [56]	
40%	3
50%	3
60%	4
70%	7
80%	3
Modified Charlson Comorbidity Index Score*	
2	2
3	5
4	5
5	4
6	3
8	1

*Charlson Comorbidity Index [57] modified for renal patients to exclude renal diagnosis and age from the scoring [58]

### Results

Findings are presented in four themes: 1. Participant views and experiences of staff-patient communication; 2. Information provision (sub-themes: receiving information from staff; missing conversations and gaps in information provision); 3. Information preferences; 4. Dialysis decision-making (sub-themes: agency and preferences; reasons for treatment decision; feelings about the decision). Illustrative quotes use pseudonyms.

### Participant views and experiences of staff-patient communication

Participants described positive experiences of communicating with their healthcare providers, including General Practitioners (family doctors), renal clinicians and palliative care providers. Participants appreciated it when staff took time to communicate and answer their questions:

*“I mean it’s, it’s a very, very nice feeling to be able to go there and know… that you can ask them any question you like. It doesn’t matter how it sounds to be but to you it’s important and they answer you and they’ve got time, that’s the thing, time for you…” Walter.*

*“Maybe, if he [the nephrologist] had asked me [pause]… I … kind of knew, I think, but if he had asked me, maybe, what do you think…or…what do you understand? So I could kind of hear things more slowly. But I don’t think he liked talking about it, he just wanted to get it done and out and on, quickly.”* Joseph.

Using comprehensible, unambiguous language was critical:

*“It’s been good if it is easy to understand, if it makes sense, if it is in my sort of language… not lots of medical stuff I don’t know. [Name of renal palliative nurse] is very good at that, anything I don’t understand she’ll tell me, blood tests or something.”* Dorothy.

*“I didn’t know I had a problem with my kidneys until I was at the diabetic clinic when they said they would ‘keep an eye on’ my kidneys. But that didn’t really mean much to me, keeping an eye on my kidneys, you know. And it wasn’t until I was referred to [name of nephrologist] and she explained things to me, that I realised it, it was quite serious.”* Florence.

Participants described the need for clinicians to balance being straight-forward, honest and matter-of-fact in their approach with being sensitive and personal:

*“I get the impression they don’t run away from it there [at hospice], they know you’ll die and meet that head on, like [hospice nurse], when she came she was honest and spoke about it, but not to make it worse, just to explain, what to expect and what to do.”* Charles.

*“[Palliative care] went more steady about things, gentle… Knew how to talk about things. Not a sort of blunt approach… ‘let’s get this done’, but more… ‘well, let’s find out what’s happening, is there anything we can do…what would you like to know?’ … Don’t misunderstand me, they could still talk about difficult things, what was happening, not having the dialysis and what that meant. It was just the way they did it. Gentle, and a kind of personal approach.”* Joseph.

This was contrasted with clinicians avoiding key issues or being insensitive, brusque or abrupt:

*“[Name of nephrologist] himself when I was still in the hospital, he said ‘If you’re still here in a few weeks or a few months, then I would be gobsmacked’ he said, not a phrase we liked. ‘If it lasted a year I would be surprised’ would be better.”* Charles.

Good communication was facilitated by a context of continuous care and an ongoing staff-patient relationship:

*“You feel more relaxed, I mean, knowing there’s always somebody there. There’s always somebody you can turn to, and I think that’s an ever-so-nice feeling, gives you a feeling of, a little bit of confidence in yourself I think.”* Donald’s carer.

Conversely, poor continuity of care and lack of an ongoing relationship could prevent good communication:

*“It was a bit of a problem in the beginning. (Pause). It was…er…it was not very good…I have to be honest…in the early days going back and forth for the tests and so on, it wasn’t very easy. Every time seemed to be some new doctor, usually I think it was the junior doctors, the regist… registrars are they?”* Betty.

Negative experiences of communication could have a significant impact; Joseph described being told, bluntly, he couldn’t have dialysis by a consultant he had met just once before:

*“It did worry me at the time. It upset me very much. I felt quite down, quite depressed about it. It was like being written off, in a way… like, ‘We can’t help you, so off you go, not our problem, just cope with it.’ It made me feel awful.”* Joseph.

### Information provision

#### Receiving information from staff

Most patients reported good experiences of accessing information from healthcare providers, with 17/20 saying they had good/adequate information at the time of deciding about whether to have dialysis:

*“They’ve been very good, all the way, just ask anything and they tell you. There’s never been any problems like that, or not finding out what you wanted or anything. No, they’ve been good, very good.”* Walter.

Renal nurses, supportive/palliative care nurses and nephrology consultants were the primary source of information, with leaflets/ hospital education sessions also mentioned. One patient had been referred to a pharmacist for detailed information about medicines. Outside of healthcare, patients consulted books and talked to others with kidney disease.

Participants stressed the importance of staff providing information proactively:

*“[The renal nurse] went all into it in, in, when I first went over there. I was, you know, I was really surprised, cos normally you go in to see the nurse and oh yes, they, they do what they gotta do and that’s it, but she did go in to everything and... she was so good, explained it all. Made it all so clear.”* Donald.

Two patients reported that their own forgetfulness limited their ability to retain information:

Interviewer: “*Can you tell me, have the, the doctors and nurses explained much to you about your kidney problem?”*

Lilian: “*Not really, I suppose. I don’t know. I suppose they did, but I’m so forgetful now that I forget all about it [laughing].”*

Another said information *“goes in one ear and out the other”* (Walter).

#### Missing conversations and gaps in information provision

There were two clear areas in which information was lacking: diagnosis and disease progression. Four patients reported delays in being told about their condition, knowing or suspecting that doctors knew there was a problem before they were informed:

*“They should have said to me earlier about the problem… ‘Cos they must have known about it for some time… As soon as there’s a problem, yes, I think you should know about it and if there anything you can do.”* Florence.

Patients’ hypothesised reasons for this were staff not wanting to concern or worry the patient needlessly, and avoiding/putting off the discussion, e.g. by repeating tests rather than talking about the situation:

*“I think they [junior doctors] didn’t know what to say sometimes, and it often seemed to be a case of ‘Well, let’s see next time.’ There is only so many times you want to hear that. I’m not silly… I know how to put two and two together…and I was beginning to work things out. But it took them a long time to tell me, really. I don’t know if it was that they didn’t really know for a while, or they didn’t want to say, but I felt it wasn’t good, no.”* Betty.

Not being told about their condition in a timely manner had negative consequences as patients couldn’t adapt their diet and health behaviour, e.g.

Karif: “*I was upset…at the beginning, yes.”*

Interviewer: “*Mmhm……what upset you?”*

Karif: “*That I was sick… I was sick without knowing… That I was doing things I was not supposed to do.”*

However, knowledge was acknowledged to have both positive and negative consequences, which could be hard to anticipate. Karif later said that, on reflection, knowing about his kidney disease hadn’t changed his behaviour and had caused worry, so he wished, despite the advantages of knowing, that he hadn’t been told at all. One patient reported that not being told earlier in the disease trajectory had upset his wife, although he did not mind himself:

*“It bothered her a lot. Not me. I think… if you need something done then they’ll do it, won’t they? I reckon I didn’t need anything before then. The wife thinks different… she says if they can’t tell you, if it takes seven years for someone to tell you what is happening, and they say, well they can’t do nothing about it, but then all of a sudden they start sending you all these letters to go to [the renal unit] to see a nephrologist … she says they ought to have done something before.”* Michael.

The second gap in information related to what to expect as disease progressed and death approached. Renal clinicians reportedly had difficulty dealing with the uncertainty of the condition and answering questions about disease progression; participants contrasted this with the more straight-forward approach of hospice staff:

Charles: *“In the background of my mind all the time is this wish to know a little bit more about the next phase… I do understand that they can’t say nearer than so many weeks, so many days but it leaves you, it leaves us both in a strange limbo which the world, the world becomes unreal after, you just have no idea but it becomes so much part of you that you forget to say it.”*

Interviewer: *“And is, is that something you’ve been able to ask professionals about?”*

Charles: *“Been able to ask but they’ve always been very skilled in not telling you. Except [name of hospice nurse], she was honest, but none of the others. Most of them, well there’s kind of a lot of hedging… they just don’t know, and aren’t able to talk about not knowing very well.”*

Reported consequences of lacking information about disease progression were that patients did not know what symptoms or problems were attributable to their renal disease, and worried about what would happen (including where they would be cared for) as they became more ill.

### Information preferences

Participants’ attitudes to information varied. In general, they wanted information about their treatment options and renal disease:

Interviewer: *“Yeah, some people prefer to have more information and some people prefer to have less. What about you?”*

Charles: “*Oh yeah, obviously more, more. The more I understand, the better. I always have wanted to know what’s going on, to try and understand – it helps you to know what you can do, doesn’t it?”*

However, there were exceptions and caveats. Betty said she did not want in-depth information about dialysis from the renal nurse as she had already decided not to have dialysis:

*“I said to [renal nurse], ‘Don’t tell me about all this dialysis, will you, because I don’t want to hear about it’. I’d already made up my mind, so what was the point. But she was trying to be sure I understood what I was turning down, so that’s fair enough.”* Betty.

Joseph said that although he wanted information, how it was conveyed was just as important as the content/amount of information. He appreciated that some clinicians tailored information and asked what information was wanted. Florence recognised the clinical challenge of tailoring information provision:

*“They have to tell you, unless of course… they know their patients and they can say ‘Well, I don’t think it’s a good thing to tell this patient ‘cos she could get hysterical and throw a wobbly’… but for me I think I would have liked to have been told. They should have told me sooner. But then perhaps other people wouldn’t like to be told. I don’t know what the answer to that is, at least not for everyone.”* Florence.

All except one patient spoke about their poor prognosis, either openly or implicitly. But several were ambivalent about receiving prognostic information, or preferred not to know details. Harold expressed the dilemma that information about the end of life raised for him:

*“It gets to a point – certain things really it’s probably best not to know, but I don’t know. Sometimes I don’t ask, because it makes you worry, but then you still worry if you don’t know.”* Harold.

Two participants talked about the limitations of health professionals’ own knowledge:

Michael: *“One of the things I’d like to know is how long my kidneys will last but they can’t tell you that exactly, and they said four or five years, so that’s not their fault is it? I want to sit down and get real answers, but the answers aren’t always there, are there?”*

Interviewer: “*That can be difficult. What do you think?”*

Michael: *“Sometimes, you get the feeling that they don’t know either… So if I ask about the kidney that’s one thing, but then if I ask something more … difficult [pause] … like what’s going to happen to me when… that, they don’t know the answer to… but they don’t always say.”*

Clinicians and patients were described as *“in the hands of a mystery”* (George); for example, George wanted to know what caused his terrible itching, Alice worried about getting water on her lungs, and Michael felt renal clinicians didn’t understand his other health problems or their interaction with his renal disease (“*it’s like they know one bit of you, but not the other bits, if you know what I mean*”).

Four participants were clear that details about the future, especially details of how death might be, were not something they really wanted to discuss. Mildred was very definite that she would rather not know:

*“I suppose kidney trouble is the same as any other trouble, eventually it takes over you and you die. This is, this is the truth of it… I went up and they said, ‘You want to know what’s going to happen in the end?’… No, no I don’t.”* Mildred.

Three other participants were more ambivalent:

*“I haven’t asked, and nobody’s said anything, is, how will the end arrive, you know. Will it be painful? Will it be a nuisance … or will it just, you know, fade away? I don’t know…”*

*“I’m wondering, you know, what, you know, will I be so doddery I won’t be able to move or make a cup a tea or something… that’s the only thing that worries me… I’m not sure that I want to know. I wonder sometimes whether I should, but I think, on balance, I’d rather not know what the future holds.”* Jack.

In contrast, Dorothy reported that she encouraged open and honest discussion of death and dying by being frank and direct herself:

*“I think it helps if you’re straight with them, then they’re straight with you… Makes it easier all round… they know that I want to hear what’s what even if I am [age 80-84]. And if that’s how I am, then it is easier isn’t it, for them to tell me what’s going on, no fussing. And we’ve talked about dying… what might happen and when.”* Dorothy.

### Dialysis decision-making

#### Agency and preferences

Of 20 participants, 16 reported that the decision not to have dialysis was their own:

Interviewer: “*Who made the decision?”*

Betty: “*Ohh… I did, I did. Yes, I was quite clear. Dr [nephrologist] said I could have dialysis if I wanted it, but I couldn’t see the point.”*

Several reported that their decision was influenced or reinforced by clinicians’ recommendations, and that this was reassuring, e.g.

*“Some of the doctors that I talk to… say, well if it had been them they’d have probably made the same decision, so that reinforces the, the decision I made was right, and I made it on my own.”* Jack.

Dorothy said her clinicians had wanted to give her dialysis and had been *‘surprised’* by her decision. Grace reported that she had encountered resistance from clinicians:

Interviewer: “*So did you make that decision or did the doctors?”*

Grace: “*I say no. [Name of daughter] she say no. Doctors say yes [laughs].”*

Interviewer: “*So you made it clear to them what you wanted?”*

Grace: “*Yeah, yeah. Doctors say to me I be dead by Christmas without it. Am I dead? [Laughs]. No, no. no. They don’t know [laughs].”*

Four patients reported the treatment decision was their doctor’s rather than their own. Two of these (Donald and Mildred) could not have dialysis due to existing comorbidities, and one (Joseph) because of problems with inserting a fistula. The two patients with comorbidities agreed with the treatment decision and had not wanted dialysis:

Donald: *“No, I didn’t want it [laughs]. Didn’t want the dialysis. I was better off out of it and I, I quite agreed with what [name of renal doctor] was saying... he was the one that says it wouldn’t be a good thing for me, and I thought well if they can do something without dialysis, I mean it’s not broken, don’t interfere, sort of thing, you know…”.*

Interviewer: “*And who made the decision would you say?”*

Donald: “*Oh, the doctor did, [name of renal doctor] did alright, he made it all clear, but I agreed with him… I’d say if I didn’t [laughs].”*

One of the patients with comorbidities said she preferred that the doctor had made the decision for her, as it meant the responsibility was not solely her own:

*“If I’d had to… then it would be me, wouldn’t it? I mean it would be me deciding and me feeling responsible for things, like if it was right or not, and I don’t know do I? Really I don’t. It’s much better if the doctor says it, then it’s not down to me like, and I don’t worry.”* Mildred.

Several patients talked about family members’ positive involvement in the decision, e.g. attending appointments to ask questions, helping to make the decision, supporting the decision made, and taking responsibility for enacting the patient’s wishes:

*“I made it on my own, I came back and told the boys [his sons] what was happening and, and they just said, ‘Well it’s your life and your decision, we’ll back you whatever you do,’ you know, and that was it.”* Harold.

One patient reported that his decision not to have dialysis had met with resistance within his family:

*“My wife wanted me to have it, she said, ‘You’ve got to have it, you just can’t not have it.’ But I told her, it’s up to me, and I want to live ‘til I die, not stop in a taxi or sat in the waiting lounge half my life.”* Michael.

#### Reasons for treatment decision

The most commonly cited reason for choosing not to have dialysis was its effect on quality of life:

Interviewer: “*And what was it about dialysis that really didn’t, you didn’t feel was right for you?”*

Charles: “*It was a prison sentence, this terrible joining of a club that you knew had only one door… I wasn’t surprised at all that they had to work like hell to keep your spirits up.”*

Patients recognised the commitment and impact of travelling to and from hospital:

*“It was the travelling to and from London three times a week for the dialysis and then it was for four or five hours, whatever it took.”* Colin.

Seven patients described how they drew on knowledge of other people who had dialysis in judging what its impact could be and deciding not to have it:

*“She [friend] died anyway. She died although she had dialysis, anyway. So what’s the point of that then? See what I mean… She used to have to be in at a certain time because she had it at home or something… And she could never go anywhere because she had to be back at certain time, yeah. I just couldn’t be bothered with that [laughing]*.” Lilian.

The complex logistics of home dialysis were also discussed:

*“She showed me the bottle of stuff and I thought… where am I going to put all that?... I’ll have all that all in my bungalow? That’s the sort of thing that I would think of.”* Mildred.

Elderly patients raised the idea of a natural lifespan coming to an end, wishing to avoid medical intervention and not use resources:

*“I’d have to go into hospital and have an operation, general anaesthetic to have this thing put into you, in your arm, or whatever it is and then I didn’t want that either. Not at my age… it is better to go with all your dignity, doing things your way, not with tubes and machines and other people dictating… it makes it all on their terms, not my terms.”* Florence.

Some patients reflected that if their spouse were still alive they might have decided differently.

#### Feelings about the decision

Four participants reported feeling low/depressed around the time the treatment decision was made. One had chosen to have conservative care himself, but then had doubts before resolving these in further discussion; two were medically advised conservative management (given co-morbidities); and the fourth (Joseph) had no option because of dialysis access problems. For Joseph, this decision was not communicated well, and he described his reactions to this, especially to being given a prognosis of only two months, but also how his feelings of depression subsequently lessened:

*“Mentally, I was very depressed when they told me I couldn’t have the fistula… that was ten months ago, so I was very depressed then, but I didn’t agree with it, certainly it wasn’t right what they said about the two months to go… but now, I don’t know how far I’m going to go.”* Joseph.

## Discussion

This study provides an in-depth description of communication and information provision among patients with ESKD receiving conservative care, and their views of the treatment decision. Participants reported variable quality in staff-patient communication and gaps in the information provided. Participants described renal clinicians avoiding, struggling with or failing to have conversations with them about diagnosis and disease progression. Consequences of this lack of communication included patients not being able to adapt their diet/health behaviour earlier in their illness, not knowing what symptoms or problems were attributable to their renal disease, and worrying about what would happen in future. This lack of knowledge compounded the challenges of living with the uncertainty of CKD5 [30].

Previous studies have reported poor communication and avoidance of discussions of diagnosis and prognosis by renal clinicians [31, 32], associated with lack of confidence and ability in breaking bad news and discussing end of life concerns [33, 34]. Prognostic uncertainty threatens healthcare providers’ self-perceptions as competent professionals, acting as a barrier to discussions with patients [35]. Other barriers include a focus on curative and technological aspects of patient care that may overlook psychosocial needs [36], feeling unprepared for discussions and uncertain of the best way to have them [37, 38], and a misperception that these conversations cause harm [39]. In fact, evidence suggests that patient experiences are better when uncertainty is shared and explained [40, 41]. In our study, participants contrasted the avoidance behaviour they encountered with the collaborative and straight-forward approach of palliative care providers and other renal clinicians.

Participants’ information needs were nuanced and changed over time. In general, participants wanted information about their treatment options and renal disease, but there were exceptions and caveats. The preferred amount of information depended on the topic, and there was ambivalence about receiving prognostic information and details about the next phase of illness and death. Other qualitative studies have also reported variation and ambivalence in patients’ preferences regarding receiving information and decision-making [31, 42]. In contrast, North American surveys report that patients with renal disease want information about end-of-life issues, including dialysis withdrawal and prognosis [43–45], with a Canadian study reporting that 95% would like their physicians to disclose life-expectancy information without prompting, even if prognosis is poor [43]. Our findings suggest that the situation is more complex and nuanced than survey data imply.

We found that patients who were clinically ineligible for dialysis sometimes felt relieved that the responsibility had been taken out of their hands, but not having a choice could also be associated with feeling depressed around the time of the decision. For other patients, the decision to have conservative care was commonly framed as a decision against dialysis, hence how dialysis was perceived was central to patients’ decision-making. Dialysis was perceived to be not a cure but a *“make do thing”,* a *“nuisance”* provided on clinicians’ *“terms”.* As in Morton et al’s systematic review [46], our participants were more concerned with the impact of treatment on their quality of life than on longevity: they were not prepared to undertake the rigours of dialysis, and perceived its negative impact to outweigh potential benefit. Participants’ perceptions of dialysis as disrupting and restricting life echoed those reported in Llewellyn et al’s study of older people with CKD5 who had declined dialysis [32]. They described logistical challenges, for example the need to travel to/from the hospital, which have previously been reported as important [31, 46]. Unlike in Morton’s review, participants had not consulted with peers to understand palliative care as an option; however, they did consider others’ experiences on dialysis when making their decision and judging what dialysis might be like.

In our study, although most patients reported that the decision not to have dialysis was their own, clinicians’ recommendations played an important role in influencing and reassuring them. This finding aligns with cohort studies demonstrating that clinicians’ views are a major factor influencing patients’ treatment decision-making [47, 48]. As also reported by Llewellyn et al. [32], we found evidence of renal clinicians resisting a patient’s decision not to dialyse. While family members often played a supportive role, the treatment decision could lead to conflict. Our older group of patients also discussed their wish to respect the natural life span, avoid medical intervention, and not use up resources, supporting Australian [49] and UK [32] findings.

This study has strengths and weaknesses. The study sample represents a maximum variation sample, with regard to age, living situation, levels of co-morbidity and functional status, which adequately reflects the variability within this population. Most participants in the study were of white ethnicity, reflecting the UK population and the catchment population for the three renal units. However, patients from minority ethnic groups might have different experiences, views and preferences regarding discussions of treatment and end of life care [50]. Further interviews with individuals from Black, Asian and minority ethnic communities would have increased the transferability of findings. The sample of 20 enabled in-depth analysis while achieving sufficient information power (including data saturation) to meet the study aims [51]. However, this study did not include clinicians or informal caregivers, who play an important role in supporting older patients and their decision-making [46], particularly when patients experience cognitive impairment. The data were collected in 2007, when the recruiting sites were actively interested in or were developing conservative management services. At other renal services at that time the culture of care might have been less supportive of conservative care; hence our findings might underestimate gaps in information and the extent to which staff avoided/struggled with discussing end-of-life issues. Since 2007 conservative care has become a more accepted component of renal care. While it is possible that this has led to improvements in communication, there is recent evidence of problematic practice; for example, the 2017 UK Renal Registry patient experience survey found that communication and clinicians’ decision-making support were highly variable across renal units, with decision-making support rated lowest of all 13 service areas [15]. This problematic practice, together with the ongoing lack of research exploring the views and experiences of patients receiving conservative care, means our findings are still highly relevant.

Our findings have implications for clinical practice, education and research. They support the hypothesis that renal clinicians’ perceptions and recommendations regarding treatment influence patients’ decision-making. To further inform education, research is needed to better understand how the way healthcare providers communicate with patients and caregivers, and the information provided, influences the process and experience of decision-making. This includes research into how the use of information leaflets, decision aids and other shared decision-making tools (https://www.england.nhs.uk/rightcare/shared-decision-making/) influence patients’ experiences of communication and decision-making and its outcomes. This evidence is crucial to support renal clinicians in the complex work of tailoring how they communicate with patients, provide information, and support treatment decision-making.

Collaboration between palliative care specialists and renal clinicians is essential in providing high-quality ESKD care, and palliative care clinicians play an important role in supporting renal clinicians’ communication with patients. However, conversations about the uncertainties of renal disease and treatment options need to occur upstream in the disease trajectory, hence renal clinicians must be well-equipped to manage them. Eliciting patients’ information-, communication- and decision-making preferences in a sensitive and ongoing manner, and tailoring care accordingly, is critical to patient-centred care. Assessment tools can assist with this process [52] (see https://pos-pal.org). However, renal clinicians struggle with the complexity of the communication required of them [11] and want training on how to discuss conservative management [53]. Patients also want improvements in how renal clinicians communicate and support their decision-making [15], as reflected in our findings. We recommend that renal clinical training and education includes how to share and explain the uncertainty of kidney disease, consequences of not discussing diagnosis/prognosis, nuances in information preferences and how to elicit these, tailoring information to be patient-centred, how clinicians’ framing of information influences patients’ decision-making, and responding to patients’ stated treatment decisions. Education which is based on direct observational evidence [54] and incorporates discussion of real patient-clinician consultations [55] may be most effective at changing clinician behaviour [55].

## Conclusions

Older patients report variable quality in communication with clinicians and gaps in the information received. Uncertainty about the disease trajectory and patients’ ambivalence regarding information makes communication particularly challenging for clinicians. Findings suggest that: (1) eliciting patient information preferences, tailoring information accordingly and conveying it clearly and sensitively is critical; and (2) nephrology training should include how to elicit patient decision-making preferences and values and make a CKD treatment recommendation that aligns with them. Our findings can inform support and training for renal clinicians to ensure decision-making support for older patients is patient-centred.

## Abbreviations

CKD: Chronic kidney disease
eGFR: Estimated glomerular filtration rate
ESKD: End stage kidney disease
